# Editorial: Advances in the Endocrine Role of the Skeleton

**DOI:** 10.3389/fendo.2020.591085

**Published:** 2020-10-15

**Authors:** Roberto Baldelli, Amelie E. Coudert, Andrea Del Fattore

**Affiliations:** ^1^Endocrinological Oncology, Service of Endocrinology, A.O. San Camillo-Forlanini, Rome, Italy; ^2^BIOSCAR, Unité Mixte de Recherche 1132, Institut National de la Santé et de la Recherche Médicale, Hôpital Lariboisière, Paris, France; ^3^UFR d'Odontologie Garancière, Université de Paris, Paris, France; ^4^Bone Physiopathology Research Unit, Genetics and Rare Diseases Research Division, Bambino Gesù Children's Hospital, Istituto di Ricovero e Cura a Carattere Scientifico (IRCCS), Rome, Italy

**Keywords:** bone, endocrine, osteocalcin, lipocalin 2, osteoclast

In the last two decades, bone tissue has been recognized as an endocrine organ able to regulate the physiology of the whole body. Indeed, from the ancient “vision” of the skeleton as only a scaffold for the body, unexpected functions associated with the bone have been discovered. The bone is now described as an endocrine organ that secretes many hormones, including FGF-23 (Fibroblast Growth Factor), Osteocalcin and Lipocalin 2, controlling whole body physiology (1).

The skeleton regulates calcium phosphate balance, energetic metabolism, insulin secretion, male fertility, muscle activity, cognitive functions, and appetite (1–4). The latter discovered function even correlates with the regulation of the “fight-or-flight” mode by osteocalcin (3). However, most of the endocrine functions of the skeleton have been extensively studied in animal models and further studies are required to confirm this regulation in humans too (1, 2).

Osteocalcin is a small protein secreted by osteoblasts and embedded in the bone matrix. During the bone resorption activity of osteoclasts, the generation of an acid compartment in Howship's lacuna reduces the carboxylation status of osteocalcin. Undercarboxylated osteocalcin is then released in the blood and it regulates insulin secretion and energy expenditure (2). Cipriani et al. described the interplay between bone and glucose metabolism. Indeed, experimental data and clinical studies have analyzed the detrimental effect that the perturbation of glucose metabolism has on the skeleton and it has been demonstrated that bone modulates glucose homeostasis. Zhang et al. demonstrated how high glucose suppresses osteogenic differentiation and semaphorin 3a, released by osteoblasts, may take part in this process. The application of exogenous semaphorin 3a alleviates the high glucose-induced inhibition of osteoblast differentiation in diabetic osteopathy.

Wang et al. reported that the large N-mid fragment of osteocalcin (N-MID) is negatively associated with probable non-alcoholic steatohepatitis, and β-CTX (β-C-terminal cross-linked telopeptides of type I collagen) and P1NP (procollagen type I N-terminal propeptide) are positively correlated with the probable presence of significant fibrosis in postmenopausal women with type 2 diabetes.

The interplay between fat tissue and bone was described by Fintini et al., analyzing how both adipocytes and osteoblasts derive from multipotent mesenchymal stem cells (MSCs) and the relationship between osteopenia and obesity. In particular, this study describes the development and growth of the bone, and how this process is influenced by physical activity, nutrition, and adipose tissue.

Moreover, osteocalcin regulates male fertility as revealed in the paper by Yang et al.. This study describes how osteocalcin is also associated with testosterone secretion and testicular volume in pathological conditions including Idiopathic hypogonadotropic hypogonadism. The extraordinary functions of this new hormone are mediated by interaction with two receptors belonging to the class C subfamily of GPCRs (G protein-coupled receptor) as Gprc6a and GPR158 (1).

Another recently described bone hormone is lipocalin 2, secreted by osteoblasts. Lipocalin 2 enters the blood and crosses the blood-brain barrier; it then binds to the Melanocortin 4 receptor (MC4R), expressed in the paraventricular and ventromedial neurons of the hypothalamus and activates an MC4R-dependent anorexigenic (appetite-suppressing) pathway (4).

The interplay between bone and other organs is regulated by secreted factors but also by extracellular vesicles that are released from the bone. The review by Li et al. described some mechanisms of this regulation mediated by extracellular vesicles. It highlighted how extracellular vesicles can transport osteocalcin and lipocalin 2 and be also involved in paracrine communication inside the bone microenvironment.

This special issue should is of interest to basic and clinical researchers since it covers a wide range of topics regarding endocrine bone research. Original studies and reviews have been published with the aim of reporting and summarizing the latest findings on the relevance of the skeleton in the physiology of the whole body.

## Author Contributions

RB, AEC, and AD wrote and revised the paper. All authors contributed to the article and approved the submitted version.

## Conflict of Interest

The authors declare that the research was conducted in the absence of any commercial or financial relationships that could be construed as a potential conflict of interest.
